# Non-Coding RNAs: Regulating Disease Progression and Therapy Resistance in Hepatocellular Carcinoma

**DOI:** 10.3390/cancers12051243

**Published:** 2020-05-15

**Authors:** Debashri Manna, Devanand Sarkar

**Affiliations:** Massey Cancer Center, Department of Human and Molecular Genetics, VCU Institute of Molecular Medicine (VIMM), Virginia Commonwealth University, Richmond, VA 23298, USA; Debashri.Manna@vcuhealth.org

**Keywords:** hepatocellular carcinoma, non-coding RNA, therapy resistance

## Abstract

Hepatocellular carcinoma (HCC), the primary liver cancer arising from hepatocytes, is a universal health problem and one of the most common malignant tumors. Surgery followed by chemotherapy as well as tyrosine kinase inhibitors (TKIs), such as sorafenib, are primary treatment procedures for HCC, but recurrence of disease because of therapy resistance results in high mortality. It is necessary to identify novel regulators of HCC for developing effective targeted therapies that can significantly interfere with progression of the disease process. Non-coding RNAs (ncRNAs) are an abundant group of versatile RNA transcripts that do not translate into proteins, rather serve as potentially functional RNAs. The role of ncRNAs in regulating diverse aspects of the carcinogenesis process are gradually being elucidated. Recent advances in RNA sequencing technology have identified a plethora of ncRNAs regulating all aspects of hepatocarcinogenesis process and serving as potential prognostic or diagnostic biomarkers. The present review provides a comprehensive description of the biological roles of ncRNAs in disease process and therapy resistance, and potential clinical application of these ncRNAs in HCC.

## 1. Introduction

### 1.1. Hepatocellular Carcinoma (HCC)

Hepatocellular carcinoma (HCC) is the most common type of primary liver cancer in adults [1]. Worldwide, it has emerged with high mortality rate in people with cirrhosis and is the second leading cause of cancer-related deaths in males [2]. Currently available HCC treatment options are curative resection, liver transplantation, radiofrequency ablation, transarterial chemoembolization, radio-embolization, and targeted therapy using sorafenib and other tyrosine kinase inhibitors (TKIs) [3]. Despite of the availability of several treatment modalities, the incidence rate of HCC has been escalating worldwide over the last 20 years due to limited therapeutic options for advance stage patients, development of chemo- and radio-resistance and recurrence of the disease [4]. As such, significant efforts are being made to unravel the mechanism underlying HCC development, progression, and chemoresistance in order to develop novel, effective and targeted therapies. Among other important factors, the role of regulatory non-coding RNAs (ncRNAs) as potential targets for HCC therapies is a promising area of research [5]. As yet, the functions of many ncRNAs are not completely recognized. However, several ncRNAs are involved in gene expression regulation, epigenetic modification, and signal transduction in both normal and cancer cells [6]. Dysregulation of these different ncRNA subtypes has been implicated in the pathogenesis and progression of many major cancers including hepatocellular carcinoma [7]. The present review provides an overview of current findings of ncRNAs function in HCC progression and therapy resistance.

### 1.2. Non-codiding RNAs

Non-coding RNAs are an abundant group of RNA transcripts that do not translate into proteins; instead, they make potentially functional RNAs [8,9]. The Encyclopedia of DNA Elements (ENCODE) project identified that 80% of the human genome transcribes ncRNAs [10]. Depending on their functions ncRNAs can be divided into two main types: infrastructural and regulatory ncRNAs [9]. Infrastructural ncRNAs, such as ribosomal, transfer and small nuclear RNAs, have a housekeeping role in protein translation and messenger RNA (mRNA) splicing. Regulatory ncRNAs are engaged in the modification of other RNAs and as such are important in epigenetic regulation [9,11]. These ncRNAs that are involved in epigenetic processes can be divided into two major groups, the short ncRNAs (<30 nucleotides) and the long ncRNAs (>200 nucleotides), and both play a role in regulating heterochromatin formation, histone modification, DNA methylation targeting, and gene silencing. Apart from their function, ncRNAs can be simply classified on the basis of their molecular size. ncRNAs longer than 200 nucleotides are designated as long ncRNAs (lncRNAs), whereas ncRNAs shorter than 200 nucleotides are regarded as small ncRNAs (sncRNAs) [7]. Small ncRNAs represent a comprehensive regulatory RNA species responsible for modulating a spectrum of gene expression, and include two categories according to their size [8]. Small-sized ncRNAs are 17–30 nt in length, such as microRNAs (miRNAs), short interfering RNAs (siRNAs), piwi-associated RNAs, and transcription initiation RNAs. Middle-sized ncRNAs are 20–300 nucleotides in length, and mainly consist of small nuclear RNAs (snRNAs), small Cajal body-specific RNAs (scaRNAs), and small nucleolar RNAs (snoRNAs) [8]. In this review, the function of regulatory ncRNAs in hepatocarcinogenesis will be discussed.

## 2. Long ncRNAs (lncRNAs) in HCC

Long ncRNAs (lncRNAs) are >200 nucleotides in length. They can be categorized according to their properties, such as transcript length, genomic location and context, sequence and structure conservation, effects on DNA sequences, functional mechanisms and targeting mechanisms, and association with protein coding genes or subcellular structures [12,13]. lncRNAs play important role(s) in the pathogenesis and progression of human cancers, including HCC [7,14]. They are involved in the regulation of proliferation, migration, apoptosis, cell cycle, tumorigenesis, and metastasis in HCC [14]. In this review we focus on those lncRNAs for which substantial literature from multiple laboratories exists delineating clinical significance and potential molecular mechanisms.

### 2.1. Long Non-coding RNAs Upregulated in HCC

#### 2.1.1. Hox Transcript Antisense Intergenic RNA (HOTAIR)

Hox transcript antisense intergenic RNA (HOTAIR), located within the Homeobox C (HOXC) gene cluster on chromosome 12, is a 2158 nucleotide lncRNA that regulates epigenetic gene silencing by functioning as a scaffold for polycomb repressive complex 2 (PRC2) and lysine-specific histone demethylase 1 (LSD1) and functions as an oncogene in many cancers [7,15]. HOTAIR was shown to be overexpressed in human HCC tissues compared to adjacent non-HCC tissues, and cumulative recurrence-free survival was significantly lower in patients with high HOTAIR levels compared to those who had low HOTAIR levels [16,17]. RNA immunoprecipitation (RIP) assay identified interaction between HOTAIR and enhancer of zeste homolog 2 (EZH2), a component of PRC2, resulting in downregulation of miR-218 and upregulation of its target the oncogene Bmi-1 [17]. Knocking down HOTAIR in HepG2 and Bel7404 human HCC cells resulted in inhibition of in vivo tumorigenicity and in vitro cell cycle arrest that was associated with activation of p14^ARF^ and p16^Ink4a^ signaling [17]. In Huh7 cells, it was documented that HOTAIR sponges miR-23b-3p, which results in upregulation of miR-23b-3p target zing-finger E-box-binding homeobox 1 (ZEB1) and a subsequent increase in epithelial-to-mesenchymal transition (EMT), invasion, and migration [18]. Recently, HOTAIR has been shown to promote exosome secretion by HepG2 cells by regulating expression and localization of multiple proteins, such as RAB35, member RAS oncogene family (RAB35), synaptosome associated protein 23 (SNAP23) and vesicle associated membrane protein 3 (VAMP3), which regulate exosome secretion process [19]. RIP assay identified interaction of HOTAIR with RAB35 protein. However, even though exosomes are known to promote cancer metastasis, the functional consequence of increased exosome production by HOTAIR was not studied. Additional targets of HOTAIR, identified in HepG2, Bel-7402 and Huh7 cells, include RNA binding motif protein 38 (RBM38), miR-145, and miR-122, promoting cancer phenotypes [20,21,22].

#### 2.1.2. Metastasis-Associated Lung Adenocarcinoma Transcription 1 (MALAT1)

Metastasis-associated lung adenocarcinoma transcription 1 (MALAT1) is a long (~7.5 kb) transcript located in human chromosome 11q that interacts with serine/arginine (SR) splicing factors and modulates their distribution in nuclear speckles thereby regulating alternative splicing of pre-mRNA [23]. Upregulated expression of MALAT1 was found in human HCC, and it was shown that MALAT1 functions as a proto-oncogene by upregulating serine and arginine rich splicing factor 1 (SRSF1) resulting in alternative splicing of several genes, such as ribosomal protein S6 kinase B1 (RPS6KB1), leading to activation of the mammalian target of rapamycin (mTOR) signaling and Wingless/Integrated (Wnt)/β-catenin pathway [24]. It was demonstrated that SRSF1 upregulation and mTORC1 activation are essential for the MALAT1-mediated transformation of liver progenitor cells. However, the mechanism by which MALAT1 activates Wnt/β-catenin pathway was not clear. In a subsequent study, the same group demonstrated that MALAT1 promoted hepatocarcinogenesis by augmenting translation of transcription factor 7 like 2 (TCF7L2) resulting in increased glycolysis and decreased gluconeogenesis [25]. It was documented that TCF7L2 is required to mediate MALAT1-induced transformation. However, a direct interaction between TCF7L2 and MALAT1 was not studied. In HCC cells, such as HepG2, MHCC97, Bel-7402, SMMC-7721, and Hep3B, MALAT1 functions as a sponge for miR-195 resulting in upregulation of its target epidermal growth factor receptor (EGFR) with subsequent activation of Phosphatidylinositol-3-Kinase/AKT serine/threonine kinase (PI3K/AKT) and Janus Kinase / signal transducer and activator of transcription (JAK/STAT) signaling pathways, for miR-143-3p resulting in upregulation of its target ZEB1, and for miR-146-5p resulting in upregulation of its target Tumor necrosis factor (TNF) receptor associated factor 6 (TRAF6) with subsequent AKT activation facilitating proliferation and invasion [26,27,28].

#### 2.1.3. Hepatocellular Carcinoma Upregulated Long Non-Coding RNA (HULC)

Hepatocellular carcinoma upregulated long non-coding RNA (HULC), located in chromosome 6p24.3 and coding for a 482 bp transcript, was first identified by cDNA microarray as the most upregulated transcript in human HCC tissues [29]. It was shown that HBV X protein (HBX) activates HULC promoter via cAMP responsive element binding protein (CREB) and knockdown of HULC abrogated in vivo growth of HepG2 cells [30]. HULC downregulated the tumor suppressor eukaryotic translation elongation factor 1 epsilon 1 (EEF1E1/P18) by inhibiting its promoter activity [30]. HULC was shown to promote lipogenesis in HepG2 and Huh7 cells by inducing methylation of CpG islands in the miR-9 promoter resulting in silencing of miR-9 [31]. As a result, there was upregulation of miR-9 target peroxisome proliferator–activated receptor alpha (PPARA) and increase in PPARA target acyl-CoA synthetase subunit ACSL1 [31]. ACSL1-induced cholesterol production stimulated proliferation of HCC cells. Interestingly, exogenous cholesterol upregulated HULC by a positive feedback loop, which involved the activation of HULC promoter by retinoid x receptor (RXRA) [31]. HULC interacts with 5′-untranslated region (UTR) of the circadian rhythm regulating gene CLOCK and increases its expression [32]. Knocking down CLOCK inhibited HULC-induced augmentation of in vivo growth of HepG2 cells indicating a key role of CLOCK in mediating its function [32]. HULC functions as a sponge for miR-107 leading to upregulation of its target E2F transcription factor 1 (E2F1) and E2F1 target sphingosine kinase 1 (SPHK1) [33]. This cascade of events resulted in HULC-induced angiogenesis [33]. It has been shown to function as a sponge for miR-2001-3p and miR-186, resulting in increase in ZEB1 and High mobility group AT-hook 2 (HMGA2), respectively [34]. In HepG2 and Hep3B cells, HULC stabilized Sirtuin 1 (SIRT1) thus inducing protective autophagy [35]. HULC upregulated ubiquitin-specific peptidase 22 (USP22), thus abrogating ubiquitin-mediated degradation of SIRT1 [35]. It was shown that miR-6825-5p, miR-6845-5p, and miR-6886-3p, which target USP22, were downregulated by HULC [35]. Although the oncogenic function of HULC is well-established its role in physiology needs to be studied. In addition to being a sponge for miRNAs, it needs to be determined whether it interacts with protein complexes regulating key biological events.

#### 2.1.4. H19 Imprinted Maternally Expressed Transcript (H19)

H19 gene is located in an imprinted region of chromosome 11p15 near insulin-like growth factor 2 (IGF2) gene and it is expressed from the maternally inherited chromosome while IGF2 is expressed only from the paternally inherited chromosome. There are conflicting reports whether H19 functions as an oncogene or tumor suppressor gene, but recent studies suggest H19 to function as an oncogene. H19 overexpression in HCC was detected in multiple datasets, including The Cancer Genome Atlas (TCGA), and was correlated with poor prognosis [36]. It was suggested that H19 sponges miR-193b to upregulate mitogen-activated protein kinase 1 (MAPK1) to promote EMT and stem cell transformation. Interestingly H19 expression was induced in HepG2 cells by tumor-associated macrophages suggesting a potential role of inflammation in regulating H19 expression [36]. Depletion of Transforming growth factor-β receptor 2 (TGFBR2) in HCC tumor initiating cells (TIC) resulted in increased in vivo tumorigenesis and was associated with marked upregulation of H19 via SRY-box transcription factor 2(SOX2) and knocking down H19 abrogated TGFBR2-deletion-induced tumorigenesis [37]. However, direct targets of H19 were not identified in this study.

#### 2.1.5. HOXA Distal Transcript Antisense RNA (HOTTIP)

Hox genes are homeodomain transcription factors required for maintaining positional identity and HOXA distal transcript antisense RNA (HOTTIP), a 7.9 kb lncRNA located in chromosome 7p15, is transcribed from the 5′ end of HOXA locus in an antisense direction and stimulates transcription of Hox genes by interacting with WD repeat domain 5/lysine (K)-specific methyltransferase 2A (WDR5/MLL) complex resulting in histone H3 lysine 4 trimethylation [38]. Both HOTTIP and its target HOXA13 were upregulated in HCC patients and their expression levels positively correlated with metastasis and negatively correlated with overall survival [39]. miR-192 and miRNA-240 target HOTTIP and the glutaminase GLS1 was identified as a downstream target of miR-192-miR-204/HOTTIP axis [40].

#### 2.1.6. Hepatocellular Carcinoma Upregulated EZH2-Associated Long Non-Coding RNA (HEIH)

Hepatocellular carcinoma upregulated EZH2-associated long non-coding RNA (HEIH), located in chromosome 5q35, is a 1.7 kb transcript which was first identified to be overexpressed in HCC tissues compared to paired peritumoral tissues and its levels negatively correlated with cumulative survival [41]. Knockdown of HEIH abrogated while overexpression of HEIH promoted in vivo tumorigenesis of HepG2, Huh7, and Hep3B cells [41]. RIP assay identified interaction of HEIH with EZH2. Chromatin immunoprecipitation (ChIP) assay identified that HEIH increased binding of EZH2 and levels of H3K27me3 across p16 promoter resulting in silencing of this tumor suppressor [41].

#### 2.1.7. Nuclear Paraspeckle Assembly Transcript 1 (NEAT1)

Nuclear paraspeckle assembly transcript 1 (NEAT1), located in chromosome 11q13.1, encodes two transcript isoforms—NEAT1v1 (3.7 kb) and NEAT1v2 (23 kb)—that are necessary for the formation of nuclear paraspeckles which are associated with retention of specific mRNAs in the nucleus [42,43]. Using capture hybridization analysis of RNA targets (CHART), it was documented that NEAT1 and MALAT1 localize to hundreds of genomic sites, mainly overactive genes, and perform both complementary and independent functions [44]. NEAT1 is overexpressed in many cancers including HCC and functions as an oncogene [7,45,46,47]. NEAT1 inhibition suppresses proliferation, migration and invasion of HepG2 and Hep3B cells, and mechanistically, NEAT1 sponges miR-485 to increase miR-485-target STAT3, miR-204 that targets Autophagy related 3 (ATG3) (thereby promoting autophagy), and miR-139-5p to increase its target TGF-β1 [45,46,48].

Upregulation of Terminal differentiation-induced ncRNA (TINCR), small nucleolar RNA host gene 5 (SNHG5), and HCC-associated lncRNA (HCAL) has been identified in human HCC, and potential mechanisms by which they promote HCC have been implicated by in vitro studies [49,50,51]. However, more in-depth in vivo studies are required to validate these findings.

### 2.2. Long Non-coding RNAs Downregulated in HCC

#### 2.2.1. Maternally Expressed Gene 3 (MEG3)

Maternally expressed gene 3 (MEG3) is an ~1.6 kb maternally imprinted tumor suppressor lncRNA located at chromosome 14q32 that is downregulated in human HCC tissues [52]. MEG3 directly interacted with DNA binding domain of p53 protein resulting in upregulation of p53 target genes, and MEG3 overexpression induced apoptosis in HepG2 cells [52]. Methylation of MEG3 promoter by DNA methyltransferases DNMT-1 and DNMT-3B caused downregulation of MEG3, and it was documented that miR-29 upregulated MEG3 expression by targeting DNMTs [53]. Systemic administration of MEG3 by MS2 bacteriophage virus-like particles (VLPs) crosslinked with GE11 polypeptide resulted in significant inhibition of in vivo xenografts of EGFR-positive HepG2 cells thus establishing its therapeutic utility [54]. MEG-3 was shown to function as a sponge for a large number of miRNAs, such as miR-664 [55,56]. However, elucidation of the functional significance of these interactions and regulations of miRNA target genes modulating phenotype require further in-depth study.

#### 2.2.2. Growth Arrest Specific 5 (GAS5)

Growth arrest specific 5 (GAS5), located in chromosome 1q25, is downregulated in many cancers and in HCC its expression levels inversely correlated with patient survival [7,57]. GAS5 overexpression inhibits proliferation and invasion and it was shown that GAS5 regulates vimentin expression although the underlying mechanism by which GAS5 regulates vimentin was not studied [57]. GAS5 functions as a sponge for a number of miRNAs, such as miR-126-3p, and miR-182, thereby modulating their target genes and regulating migration and invasion of HepG2, HuH6, and Hep3B cells [58,59].

#### 2.2.3. Forkhead Box F1 (FOXF1) Adjacent Non-Coding Developmental Regulatory RNA (FENDRR)

FENDRR is located in chromosome 16q24 and interacts with PRC2 and Trithorax (TrxG)/MLL complexes, thus regulating epigenetic gene expression [60]. FENDRR is downregulated in HCC tissues and overexpression of FENDRR inhibited in vitro proliferation and invasion and in vivo tumorigenicity of Hep3B and HepG2 cells [61]. Glypican-3 (GPC3) is a marker of aggressive HCC with poor prognosis and FENDRR was shown to directly interact with GPC3 promoter resulting in methylation-induced silencing [61]. FENDRR functions as a sponge for miR-423-5p that targets growth arrest and DNA damage-inducible beta (GADD45B) resulting in suppression of in vivo tumorigenicity of MHCC97 cells [62]. A potential role of FENDRR in regulating regulatory T cells (Tregs) and immune escape was suggested, which requires further validation [62].

#### 2.2.4. Downregulated in Liver Cancer Stem Cells (DILC)

Downregulated in liver cancer stem cells (DILC), located in chromosome 13q34 and coding for a ~2.4 kb transcript, was cloned as a novel lncRNA downregulated in liver cancer stem cells and knocking down DILC increased in vivo tumorigenesis by these cells [63]. DILC expression was downregulated in HCC tissues compared to peritumoral tissues, and its levels positively correlated with overall survival and negatively correlated with tumor recurrence [63]. Mechanistically, DILC was shown to interact with interleukin-6 (IL-6) promoter thereby blocking Nuclear factor κB (NF-κB)-mediated oncogenic IL-6/STAT3 signaling [63].

In addition, downregulation of lncRNA ultraconserved non-coding RNA uc.134 and an X-inactive-specific transcript (lnc-FTX) has been shown in HCC, and their potential molecular mechanisms in hepatocarcinogenesis have been implicated (Table 1) [64,65].

## 3. MicroRNAs (miRNAs) in HCC

MicroRNAs (miRNAs) represent a conserved class of single-stranded ncRNAs that are 19–24 nt in length [67]. They play a pivotal role in post transcriptional regulation of gene expression typically by an interaction between the 5′ end of the miRNA with complementary sequences of target RNAs affecting their stability and translation [67,68]. miRNAs are transcribed by RNA polymerase II as capped and polyadenylated primary transcripts (pri-mRNA) that are subsequently processed by Drosha and Dicer ribonucleases to generate precursor miRNAs (pre-miRNA) and mature miRNA, respectively [69]. The mature miRNA is loaded onto RNA-induced Silencing Complex (RISC) where in most cases it binds to 3′-UTR of mRNAs to induce their degradation or repress translation [69]. However, binding of miRNAs to 5′-UTR or coding sequences have been documented as well [70,71,72,73]. For each miRNA, the complementary sequence is present in multiple genes, and as such, each has multiple targets [67,69]. As such miRNAs have the ability to affect key cellular processes, such as cell differentiation, cell cycle regulation, metabolism and apoptosis [74]. Their oncogenic and tumor suppressor roles have been demonstrated in all cancers including HCC [7]. A plethora of differentially expressed miRNAs in HCC have been identified by miRNA microarray and similar methods in a variety of cohorts of patients [75,76,77,78,79,80]. Here, we focus on those miRNAs for which comprehensive literature is available to confirm their oncogenic or tumor suppressor properties.

### 3.1. Oncogenic miRNAs

#### 3.1.1. miR-21

Located in chromosome 17q23, miR-21 is overexpressed in many cancers functioning as an oncogene [7]. miRNA microarray identified miR-21 to be the most highly overexpressed miRNA in human HCC and it was demonstrated that it augments proliferation and invasion of several human HCC cells, such as HepG2, PLC/PRF-5, SK-HEP-1, and SNU-182, by targeting phosphatase and tensin homolog (PTEN), a negative regulator of oncogenic PI3K/AKT pathway [81]. In human HCC, a positive correlation between miR-21 and high-mobility group box 1 (HMGB1) was identified, and it was shown that HMGB1 positively regulates miR-21 expression by activating IL-6/STAT3 signaling [82]. Reversion inducing cysteine rich protein with kazal motifs (RECK) and tissue inhibitor of metalloproteinase 3 (TIMP3), which promote invasion and metastasis by regulating matrix metalloproteinases (MMPs) were identified as targets of miR-21 and anti-miR-21 inhibited tumorigenicity of Huh7 cells overexpressing HMGB1 [82]. miR-21 expression was increased in the livers of high fat diet (HFD)-fed mice and knockdown of miR-21 abrogated lipid accumulation in these mice [83]. The transcriptional repressor HMG-box transcription factor 1 (HBP1) was identified as a miR-21 target resulting in an increased expression of p53 leading to cell cycle arrest and decreased expression of p53 target gene sterol regulatory element binding transcription factor 1 (SREBP1C) leading to decreased lipogenesis. It was suggested that inhibition of miR-21 could be a potential treatment strategy both for HCC and its precursor condition non-alcoholic fatty liver disease (NAFLD). Argonaute crosslinking immunoprecipitation (Argonaute-CLIP) sequencing identified the RNA interactome of miR-21 identifying novel targets, such as Calmodulin regulated spectrin associated protein 1 (CAMSAP1), DEAD-box helicase 1 (DDX1), and Myristoylated alanine rich protein kinase C substrate like 1 (MARCKSL1), the expressions of which correlated with HCC patient survival, and also identified required for meiotic nuclear division 5 homolog A (RMND5A), an E3 ubiquitin ligase, as a miR-21 target, suggesting a widespread gene expression regulation by miR-21 [84].

#### 3.1.2. miR-221

A comparison between HCC tissues with normal liver and precancerous cirrhotic liver identified miR-221 as one of the 12 miRNAs showing significant diagnostic value and overexpression of miR-221 increased tumorigenicity by p53-/-, myc-expressing liver progenitor cells [80]. miR-221 targets p27 and DNA damage-inducible transcript 4 (DDIT4), a modulator of mTOR pathway, was identified as a novel target of miR-221, although the role of DDIT4 in mediating the oncogenic functions of miR-221 was not studied [80]. Using a two-thirds partial hepatectomy model and an adeno-associated virus expressing miR-221, it was shown that miR-221 promotes liver regeneration, and a potential role of its target aryl hydrocarbon nuclear receptor (ARNT) was implicated in this process [85]. Anti-miR-221 oligonucleotide treatment significantly reduced orthotopic xenograft growth of PLC/PRF/5 cells suggesting its potential use for HCC therapy [86]. Additional targets of miR-22, identified in human HCC cells include the pro-apoptotic Bcl-2 homology 3 (BH3)-only protein BCL2 modifying factor (BMF), cyclin dependent kinase inhibitor 1C (CDKN1C/p57), and histone deacetylase 6 (HDAC6), mediating its oncogenic function [87,88,89].

#### 3.1.3. miR-155

A choline-deficient diet model of NASH-HCC identified upregulation of miR-155, along with miR-221, miR-222, and miR-21 [90]. miR-155 is induced by proinflammatory cytokines and a role of NF-κB in the induction of miR-155 was documented in this model. The tumor suppressor CCAAT enhancer binding protein beta (C/EBPβ) was identified as a target of miR-155 and overexpression of miR-155 increased growth of Hep3B and HepG2 cells. HCV infection also induced miR-155 via NF-κB and miR-155 activated Wnt/β-catenin pathway by targeting Anaphase promoting complex (APC), resulting in increased in vivo tumorigenicity [91]. Increased miR-155 expression was identified in Epithelial cell adhesion molecule (EpCAM)-positive HCC stem cells and inhibition of miR-155 abrogated in vitro cancer phenotypes in these cells [92]. Co-culture with liver cancer-associated mesenchymal stem cells (LC-MSCs) augmented in vivo tumorigenicity of MHCC97L cells [93]. LC-MSCs release S100 calcium binding protein A4 (S100A4) that stimulates the expression of miR-155 in MHCC97L and SMMC-7721 cells. By targeting Suppressor of cytokine signaling 1 (SOCS1), miR-155 activates STAT3 signaling leading to Matrix metallopeptidase 9 (MMP9) production and increased invasion [93].

### 3.2. Tumor Suppressor miRNAs

#### 3.2.1. miR-122

miR-122 is a highly abundant liver-specific miRNA accounting for 70% of the total miRNAs in the liver and is downregulated in ~70% of human HCC [76]. CyclinG1 was identified as a direct target of miR-122 [76]. Knocking out miR-122 in mice resulted in steatohepatitis and HCC with profound alterations of a plethora of genes regulating lipid metabolism, inflammation and fibrosis [94]. Adeno-associated virus (AAV)-mediated delivery of miR-122 markedly inhibited Myc-driven HCC in mice, thereby establishing both the tumor suppressor function of miR-122 and its therapeutic utility [94]. A separate group also knocked out miR-122 and observed similar phenotypes and identified the pro-fibrogenic transcription factor Kruppel like factor 6 (KLF6) as a target of miR-122 [95]. Analysis of liver transcriptome after deletion of miR-122 at multiple timepoints revealed widespread deregulation of hepatic transcription including progressive increases in expression of imprinted genes, such as those in Igf2 and Dlk1-Dio3 clusters, providing insights into the mechanism by which miR-122 functions as a tumor suppressor [96]. Argonaute-CLIP sequencing in human and mice identified novel miR-122 targets, such as B cell lymphoma 9 (BCL9), Solute carrier family 25 member 2 (SLC52A2) and Syntaxin 6 (STX6), that could predict survival in HCC patients [97]. A liver-targeted oncolytic herpes simplex virus (HSV) delivering miR-122 showed strong in vivo efficacy in Hep3B xenograft models [98]. Interestingly, miR-122 binds to 5′-UTR of HCV RNA facilitating translation and hence replication of HCV, a major cause of HCC [73]. A locked nucleic acid (LNA)-modified oligonucleotide complementary to miR-122 facilitated long-lasting suppression of HCV viremia [99]. In Phase 2a, clinical trials involving seven international sites, Miravirsen, an LNA-modified antisense miR-122, showed long-term reductions in HCV RNA levels without inducing viral resistance [100]. In this regard, in HCV-HCC patients, treated with miR-122, monitoring for HCV viremia will be essential to ensure safety. Serum miRNA analysis identified miR-122 as the most overexpressed miRNA in NASH patients compared to controls and its serum levels correlated with the stages of the disease [101]. Thus miR-122 might play variable functions in HCC predisposing conditions, such as HCV or NASH, versus in HCC itself.

#### 3.2.2. miR-29

miR-29 is downregulated in HCC and its expression levels correlate with disease free survival in HCC patients [102]. Overexpression of miR-29 resulted in apoptosis induction and marked inhibition of in vivo tumorigenicity by HepG2 cells and the anti-apoptotic molecules Bcl-2 and Mcl-1 were identified as direct targets of miR-29 [102]. Alpha fetoprotein (AFP) is a marker of aggressive HCC with poor outcome. In AFP+ HCCs, miR-29 was most significantly downregulated along with upregulation of its target DNA methyltransferase 3A (DNMT3A) resulting in increased DNA methylation and distinct global DNA methylation patterns [103]. ChIP assay identified c-Myc to bind to miR-29 and inhibit its transcription.

#### 3.2.3. miR-101

miR-101 is markedly downregulated in human HCC and it targets Mcl-1 so that its overexpression induces apoptosis and retards in vivo tumorigenicity by HepG2 cells [104]. It was demonstrated that EZH2 epigenetically silences many tumor suppressor miRNAs, including miR-101, in human HCC cells, such as SMMC-7721, MHHCC97L and HepG2 [105]. EZH2 interacts with MYC and MYC recruits polycomb repressor complex (PRC2) to miR-101 promoter to induce methylation-mediated silencing [106]. Interestingly, miR-101 inhibits PRC2 subunits EZH2 and EED creating a double negative feedback loop promoting HCC. Several oncogenes, such as Stathmin 1 (STMN1), JUNB and Chemokine (C-X-C motif) receptor 7 (CXCR7), were identified to be targets of miR-101 [106]. Systemic delivery of a lentivirus expressing miR-101 inhibited in vivo growth of LM9 cells in the liver as well as intrahepatic and distant metastasis, and along with other known targets, Rho associated coiled-coil containing protein kinase 2 (ROCK2) was identified as its target resulting in inhibition of Rho/Rac activation, EMT, and angiogenesis [107].

#### 3.2.4. The Let-7 Family of miRNAs

The let-7 family of miRNAs are one of the most extensively studied tumor suppressors especially because of their ability to target RAS [7,108]. All let-7 family members have been shown to be downregulated by HBx [109]. It was documented that let-7a targets the oncogenic transcription factor STAT3. Similarly let-7 family was also shown to be downregulated in HCV-associated HCC [110]. The let-7 family was identified as a component of a miRNA hub that are transcriptionally regulated by PPARγ and target fibrogenic genes [111]. During liver fibrosis these miRNAs are downregulated and thus were collectively termed as anti-fibrotic miRNAs. It was documented that let-7g is highly downregulated in metastatic HCC compared to non-metastatic HCC and high let-7g expression in HCC tissues versus non-HCC tissues conferred significantly increased overall survival in these patients [112]. Type I collagen a2 (COL1A2) was identified as a target of let-7g regulating cell migration [112]. In nude mice, systemic administration of cholesterol-conjugated let-7a mimics significantly inhibited the growth of orthotopic xenografts of HepG2 cells, suggesting the therapeutic potential of this approach [113].

#### 3.2.5. The miR-15 Family

The miR-15 family is comprised of miR-15a, miR-15b, miR-16, miR-195, and miR-497 all having the same seed sequence and has shown to be downregulated in human HCC [114,115]. This family directly targets I-kappaB kinase (IKKα) and TGF-beta activated kinase 1 (MAP3K7) binding protein 3 (TAB3), upstream regulators of NF-κB signaling pathway, thus playing a key role in regulating inflammation, a key contributing factor to HCC [115]. Overexpression of miR-195 induces G1/S arrest and several G1/S transition-related molecules, such as cyclin D1, Cyclin dependent kinase 6 (CDK6) and E2F3, were identified to be direct targets of miR-195 [114]. It was shown that miR-195 inhibits angiogenesis and metastasis of QGY-7703 cells by directly targeting Vascular endothelial growth factor (VEGF), Vav guanine nucleotide exchange factor 2 (VAV2), and Cell division cycle 42 (CDC42) [116].

## 4. Small Nucleolar RNAs (snoRNAs) in HCC

Small nucleolar RNAs (snoRNAs) are widely characterized ncRNAs that primarily accumulate in the nucleoli and consist of 60–300 nucleotides [117]. A subset of snoRNAs is situated in Cajal bodies, thus occasionally termed scaRNAs. SnoRNAs are mainly responsible for the posttranscriptional modification and maturation of ribosomal RNAs (rRNAs), small nuclear RNAs (snRNAs), and other cellular RNAs. SnoRNAs are divided into two classes based on their structure and function, C/D box snoRNAs and H/ACA box snoRNAs. C/D box snoRNAs guide 2′-O-ribose methylation, and H/ACA box snoRNAs direct the pseudouridylation of nucleotides [117]. snoRNAs mainly regulate ribosomal function and as such they were considered predominantly as housekeeping RNAs. However, their role in various disease processes and oncogenesis is increasingly being appreciated [118]. Like other ncRNAs, snoRNAs can function both as oncogenes and tumor suppressor genes. SNORD126 is overexpressed in human HCC and is promoted in vivo tumorigenicity by Huh7 cells [119]. Affymetrix microarray identified overexpression of Fibroblast growth factor receptor 2 (FGFR2) mRNA with subsequent activation of PI3K/AKT pathway by SNORD126 [119]. The mechanism by which SNORD126 increased FGFR2 mRNA was not studied, which is an important question because snoRNAs regulate gene expression post-transcriptionally. Additional snoRNAs, which are upregulated in HCC and promote tumorigenesis but for which the underlying mechanism is not clear, include SNORD78, snoU2_19, SNORD76, and ACA11 [120,121,122,123]. SNORA24 levels were significantly downregulated in human HCC tissues when compared to adjacent non-tumor tissues and showed inverse correlation with overall survival in HCC patients [124]. LNA-targeted SNORA24 protected from oncogenic NRAS^G12V^-induced senescence and promoted NRAS^G12V^-mediated hepatocarcinogenesis. Lack of SNORA24 function resulted in increased translational miscoding and stop codon readthroughs suggesting perturbations of ribosomal functions contributing to HCC. Promoter hypermethylation-mediated downregulation of SNORD113-1 was shown in HCC and SNORD113-1 inhibited in vivo xenografts of HepG2 cells which was associated with inhibition of MAPK/ERK and TGF-β signaling [125]. However, the molecular mechanism by which SNORD113-1 exerts these effects was not elucidated.

## 5. P-Element Induced Wimpy Testis (PIWI)-Interacting RNAs (piRNAs) in HCC

P-Element induced wimpy testis (PIWI)-interacting RNAs (piRNAs) is an important class of small ncRNA (24–30 nucleotides) previously named as “repeat associated small interfering RNAs (rasiRNAs),” which are abundant in animal cells. They interact with PIWI proteins of the Argonaute family to form RNA-protein complexes and are linked with silencing of genetic elements [126]. In cancer cells piRNAs are involved in modulation of cell proliferation, apoptosis, metastasis and invasion, and might be considered as potential prognostic and diagnostic biomarkers [127]. A very few information is available on piRNA function during liver carcinogenesis. Small RNA sequencing was used to analyse expression pattern of piRNAs at different stages during the progression of hepatocarcinogenesis identifying deregulated expression of many piRNAs in dysplatic nodules and in HCC [128]. Similar sequencing methods identified a novel piRNA, piR-Hep1, to be up-regulated in HCC that promoted proliferation and invasion potentially by modulating PI3K/AKT signaling pathway (Table 2) [129].

## 6. Circular RNAs (circRNAs) in HCC

Circular RNAs (circRNAs), formed from back-splicing circularization of exons catalysed by the spliceosomal machinery, is a type of 3′ and 5′ covalently closed ncRNAs [130]. circRNAs act as a miRNA sponge to control the function of miRNAs, and regulate RNA processing and transcription. [130]. The role of circRNAs as oncogenes or tumor suppressor genes is being elucidated in cancer, and a recent study analyzing more than 2000 clinical samples from ~40 cancer sites identified more than 160,000 differentially expressed circRNAs in cancer patients [131]. circRNA microarrays using HCC tissues or plasma have identified hundreds of differentially expressed circRNAs in HCC patients, demonstrating that circRNAs play important role in HCC development and progression and they can serve as reliable biomarkers for HCC diagnosis [132]. circMTO1 and cSMARCA5 are downregulated in HCC patients, their expression levels negatively correlate with HCC patient survival and their overexpression inhibited in vivo growth of SMMC-7721 xenografts [133,134]. cirMTO1 sponges miR-9 that targets p-21, while cSMARCA5 sponges miR-17-3p and miR-181b-5p that target TIMP3 [133,134]. The EMT-promoting transcription factor Twist1 transcriptionally regulates circ-10720 which is overexpressed in HCC and in an inducible Twist-1 expressing mouse HCC model circ-10720 knockdown inhibited tumor growth [135]. circ-10720 sponges several miRNAs targeting vimentin [135]. circMAT2B was identified to be an oncogenic circRNA that stimulated Huh7 and HepG2 xenograft growth by promoting glycolysis via sponging miR-338-3p and regulating pyruvate kinase (PKM2) [136]. circASAP1 was overexpressed in metastatic HCC patients and promoted pulmonary metastasis by PLC/PRF/5 cells in vivo. circASAP1 sponged miR-326 and miR-532-5p, increasing their targets MAPK1 and colony stimulating factor 1 (CSF1), respectively, that contributed to promote tumor cell proliferation and invasion as well as macrophage infiltration in the tumor [137]. circRHOT1 showed progressive overexpression from early to advanced HCC and its levels correlated with poor prognosis [138]. circRHOT1 knockdown abrogated in vivo tumorigenesis of Hep3B and Huh7 cells and mechanistically circRHOT1 recruited histone acetyltransferase TIP60 to Nuclear receptor subfamily 2 group F member 6 (NR2F6) promoter to increase its transcription [138]. Examples of deregulated circRNAs in HCC is shown in Table 3.

## 7. Role of ncRNAs in HCC Therapy Resistance

### 7.1. Therapy for Advanced, Nonresectable HCC

**Figure 1:**
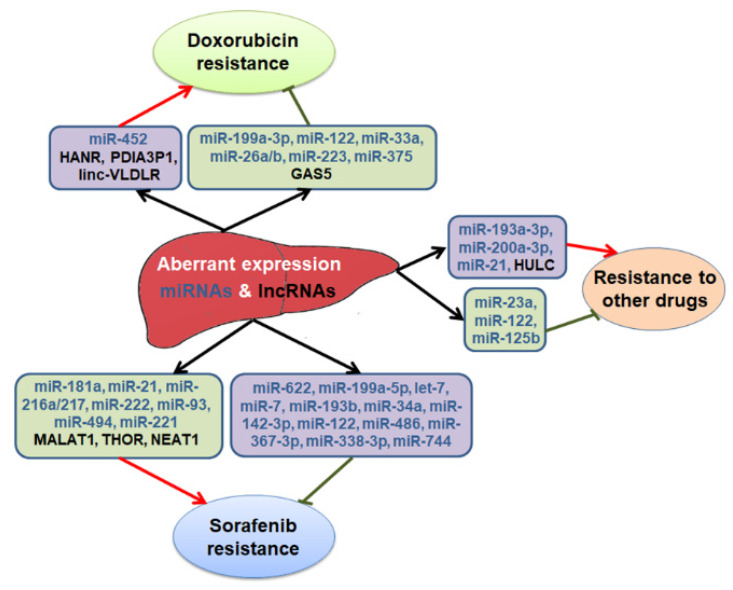
Non-coding RNAs contributing to therapy resistance in hepatocellular carcinoma HCC.

Advanced, nonresectable HCC patients are treated by targeted therapy, chemotherapy and immunotherapy [3]. Sorafenib inhibits multiple kinases—such as Raf-1, B-Raf, vascular endothelial growth factor receptor (VEGFR), and platelet-derived growth factor receptor β (PDGFR-β)—and blocks downstream MAPK and PI3K/AKT signaling pathways [139]. Raf-1 and VEGF signaling pathways play a role in the molecular pathogenesis of HCC, providing a rationale for administering sorafenib to HCC patients, and sorafenib has been the standard of care as the first line therapy for advanced HCC, following a phase III clinical trial showing survival benefits [140]. A second oral tyrosine kinase inhibitor (TKI), lenvatinib, has also been approved as first line therapy for unresectable HCC following a phase III trial [141]. Other TKIs that are being used as second line therapy in patients who have received sorafenib treatment include regorafenib, cabozatinib, and tivantinib [142,143,144]. In addition, ramucirumab, a monoclonal antibody that blocks VEGF2R signaling, is also used as second line therapy for HCC patients [145]. Systemic chemotherapy remains a crucial treatment modality for patients with advanced HCC. Chemotherapeutic drugs commonly used for HCC are doxorubicin (adriamycin), 5-fluorouracil (5-FU), cisplatin, oxaliplatin and gemcitabine as either a single agent or combination therapy [146]. A promising approach for HCC patients is immunotherapy which includes immune checkpoint blockers/monoclonal antibodies against the programmed cell death protein 1 (PD-1), PD-1 ligand (PD-L1), and cytotoxic T lymphocyte antigen-4 (CTLA-4) such as nivolumab, pembrolizumab, MED14736, ipilimumab, and tremelimumab [147]. The PD-1 inhibitors, nivolumab and pembrolizumab, have now been approved for HCC treatment as a second line therapy following sorafenib [148]. However, these treatment modalities provide very modest survival advantages, and most HCC patients develop drug resistance, resulting in poor prognosis. A potential role of ncRNAs in HCC therapy resistance (Figure 1) will be discussed next.

### 7.2. Non-coding RNAs in Sorafenib Resistance

Sorafenib is being used as the first line therapy for advanced HCC for more than a decade and as such most studies are focused on analyzing resistance to sorafenib. Several studies reported that abnormal expression of miRNAs is involved in sorafenib resistance by regulating MAPK and PI3K/AKT signaling pathways, and modulating apoptosis and autophagy [149,150,151,152]. KRAS is increased in HCC patients and activates RAF/ERK and PI3K/AKT pathways, and miR-622 is downregulated in HCC patients and it directly targets KRAS. Sorafenib resistance was associated with upregulation of KRAS and downregulation of miR-622 and a KRAS inhibitor or miR-622 mimic could overcome sorafenib resistance [150]. An in vitro study identified a role of miR-181a in sorafenib resistance of HepG2 and Hep3B cells by targeting Ras association domain family member 1 (RASSF1), a negative regulator of MAPK signaling [151]. It was demonstrated that miR-199a-5p and let-7c are downregulated in several human HCC cells and target MAP4K3 and combination of miR-199a-5p and let-7c potentiated in vitro anti-cancer effects of sorafenib [152]. However, more in-depth studies are required to determine whether these two miRNAs really play a role in sorafenib resistant HCC patients.

Phosphatase and tensin homolog (PTEN) is a negative regulator of PI3K/AKT pathway and multiple miRNAs target PTEN, and the subsequent activation of PI3K/AKT signaling results in sorafenib resistance. miRNA array between parental and sorafenib-resistant clones of Huh7 cells (Huh7-SR) identified upregulation of miR-21 in sorafenib-resistant cells [153]. It was shown that miR-21 targets PTEN resulting in activation of AKT and anti-miR-21 overcame sorafenib resistance and potentiated sorafenib-induced autophagy in vitro and in in vivo xenograft assays [153]. miR-216a/217 cluster was identified to be upregulated in recurrent HCC tissue samples and activated TGF-β and PI3K/AKT signaling by targeting SMAD family member 7 (SMAD7) and PTEN, respectively [154]. miR-216a/217 overexpression induced EMT and resistance to sorafenib. Similarly, overexpression of PTEN-targeting miRNAs, such as miR-222, miR-93, and miR-494, has been shown to increase resistance to sorafenib [155,156,157,158]. However, whether these miRNAs are increased in sorafenib-resistant cells and contribute to acquired sorafenib resistance remains to be seen. On the other hand, it was demonstrated that miR-7 is downregulated in Huh7-SR cells [159]. miR-7 targets TYRO3, a receptor tyrosine kinase, and downregulation of miR-7 resulted in activation of TYRO3-mediated activation of PI3K/Akt pathway. miR-7 overexpression resulted in significant reduction of EC_50_ of sorafenib in Huh7-SR cells by in vitro assays.

Using a rat HCC model treated with sorafenib, it was identified that miR-221 was upregulated in HCC nodules that do not respond to sorafenib compared to responders [160]. In patient sera, higher miR-221 levels before sorafenib treatment were associated with increased disease progression. Caspase-3 was identified as miR-221 target conferring resistance to sorafenib-induced apoptosis. Stable integration of HBV genome in HepG2 cells resulted in sorafenib resistance which was associated with downregulation of miR-193b and upregulation of its target Mcl-1, an anti-apoptotic protein [161]. Similarly, let-7 family of miRNAs target Bcl-xL, while miR-34a targets Bcl-2 and thus potentiate sorafenib-induced apoptosis [162,163]. Autophagy-related 5 (ATG5) and autophagy-related 16-like (ATG16L1) were identified as targets of miR-142-3p which conferred sorafenib sensitivity by decreasing sorafenib-induced autophagy and increasing sorafenib-induced apoptosis in in vivo xenograft assays [164]. miRNA microarray identified miR-122 to be downregulated in sorafenib-resistant Huh7 and PLC/PRF/5 cells and miR-122 mimic restored sorafenib sensitivity [165]. It was demonstrated that miR-122 targets IGF-1R and activation of IGF signaling was implicated to mediate sorafenib resistance [165]. In a separate study, upregulation of miR-122 target solute carrier family 7 member 1 (SLC7A1), an arginine transporter, resulting in increased nitric oxide (NO) levels was shown to contribute to sorafenib resistance [166]. miR-486, targeting Rho-interacting serine/threonine kinase (CITRON) and Claudin 10 (CLDN10), miR-367-3p, targeting MDM2 and thus activating androgen receptor signaling, and miR-338-3p, targeting Hypoxia-inducible factor -1 (HIF-1α), have been shown to increase sorafenib sensitivity [167,168,169]. Again, direct evidence of these miRNAs mediating sorafenib resistance is lacking. Exosomal miR-744, identified from HCC patient sera, was downregulated in sorafenib-resistant HepG2 cells [170]. Paired box 2 (PAX2) was suggested to be a target of miR-744. However, the mechanism by which PAX2 upregulation confers sorafenib resistance was not studied.

MALAT1 was found to be significantly overexpressed in sorafenib-resistant HepG2 and SMMC-7721 cells and overexpression of MALAT1 conferred in vitro sorafenib resistance to these cells and MALAT1 knockdown increased sorafenib sensitivity in in vivo tumorigenesis assays [66]. By sponging miR-140-5p, MALAT1 increased miR-140-5p target Aurora-A contributing to sorafenib resistance. Among the lncRNAs, THOR (testis-associated highly conserved oncogenic long non-coding RNA) has been shown to cause expansion of cancer stem cells by stabilizing β-catenin, and THOR knockdown increased sorafenib sensitivity in vitro [171]. NEAT1 contributes to sorafenib resistance by sponging miR-335 resulting in activation of c-Met-Akt pathway, and NEAT1 knockdown increased sorafenib sensitivity of xenografts of HepG2 cells [172].

### 7.3. Non Coding RNAs in Doxorubicin Resistance in HCC

Doxorubicin is an anthracycline compound that inhibits topoisomerase II hence DNA replication thereby inhibiting tumor cell proliferation. Most studies focused on ncRNAs the manipulation of which increased sensitivity of HCC cells to doxorubicin [173]. miR-199a-3p is downregulated in HCC and its overexpression in HepG2 cells increased doxorubicin sensitivity by targeting mTOR and c-Met [174]. miR-122 was downregulated in doxorubicin-resistant Huh7 cells and increased doxorubicin sensitivity by targeting PKM2, several transporters contributing to multidrug resistance, and cyclin G1 that increased p53 protein stability [175,176,177]. HepG2 cells were cultured in the presence of doxorubicin and sorafenib and the resultant chemoresistant stem-like cells, capable of generating hepatospheres and metastatic tumors in mice, showed increased expression of miR-452 which targeted SRY-box transcription factor 7 (SOX7) that inhibits Wnt/β-catenin signaling pathway [178]. Lgr5+ HCC stem-like cells having increased chemoresistance showed decreased expression of miR-33a that targets the drug transporter ATP binding cassette subfamily A member 1 (ABCA1) and miR-33a overexpression sensitized HCC xenografts to doxorubicin [179]. Doxorubicin treatment induced autophagy in HepG2 cells and downregulated miR-26a/b which inhibited autophagy by targeting unc-51 like autophagy activating kinase 1 (ULK1) [180]. A lentivirus delivering miR-26a/b could sensitize HepG2 xenografts to doxorubicin by inhibiting autophagy and promoting apoptosis [180]. miR-223 could also inhibit doxorubicin-induced autophagy by targeting FOXO3a and a combination of AgomiR-223 and doxorubicin could significantly inhibit Huh7 xenograft versus either agent alone [181]. miR-375 targets the oncogene AEG-1/MTDH, a potent inducer of chemoresistance, and miR-375 and doxorubicin, co-loaded onto lipid-coated calcium carbonate nanoparticles, markedly inhibited xenograft growth of doxorubicin-resistant HepG2 cells as well as primary tumor growth in an Akt/Ras-induced HCC model [182]. This modality of treatment exhibited less toxicity, especially cardiotoxicity, compared to free doxorubicin, demonstrating therapeutic utility.

Hepatocellular carcinoma (HCC)-associated long noncoding RNA (HANR) was overexpressed in human HCC tissues and knockdown of HANR sensitized subcutaneous and orthotopic xenografts of Hep3B and Huh7 cells to doxorubicin [183]. RIP assay identified GSK3B interacting protein (GSKIP) to interact with HANR resulting in increased phosphorylation of GSK3β [183]. However, whether this mechanism contributes to doxorubicin sensitivity was not studied. lncRNA PDIA3P1 (protein disulphide isomerase family A member 3 pseudogene 1) was upregulated in HCC, and its expression levels correlated with poorer recurrence-free survival [184]. PDIA3P1 induced doxorubicin resistance both in vitro and in vivo by binding to miR-125a/b and miR-124 that targets TRAF6, leading to activation of the NF-κB pathway [184]. Doxorubicin induced PDIA3P1 levels by inhibiting interaction between PDIA3P1 and RNA degradation protein hMTR4 (human homologue of mRNA transport mutant) [184]. GAS5 levels were downregulated in doxorubicin-resistant HepG2 cells and GAS5 overexpression sensitized xenografts of these cells to doxorubicin [185]. GAS5 functioned as a sponge for miR-21 resulting in increased PTEN levels. Treatment with sorafenib, camptothecin and doxorubicin induced expression of extracellular vesicle long noncoding RNA (linc-VLDLR) which was upregulated in HCC and its knockdown ameliorated chemoresistance by reducing ATP binding cassette subfamily G member 2 (ABCG2) [186].

### 7.4. Non-coding RNAs Conferring Resistance to Other Chemotherapeutic Agents

In 5-FU-sensitive QGY-7703 cells the levels of miR-193a-3p were downregulated via promoter methylation [187]. In 5-FU-resistant SMMC-7721 cells increased miR-193a-3p resulted in decreased levels of its targets E2F1 and the splicing factor SRSF2 which facilitates generation of pro-apoptotic splicing variant of caspase-2. AntagomiR of miR-193a-3p sensitized HCC xenografts to 5-FU [187]. A lentivirus-mediated library screening identified overexpression of miR-200a-3p to be overexpressed in 5-FU-resistant Hep3B cells. miR-200a-3p conferred resistance to 5-FU, doxorubicin and cisplatin and targeted dual-specificity phosphatase 6 (DUSP6) [188]. Overexpression of miR-23a sensitized HepG2 cells to topoisomerase inhibitor etoposide both in vitro and in vivo by targeting topoisomerase 1 (TOP1) [189]. miR-21 provided cisplatin resistance to HepG2 and Huh7 cells by targeting Fas ligand [190]. miR-122 downregulation was associated with activation of Wnt/β-catenin pathway and upregulation of MDR1, and AgomiR-122 could sensitize Bel-7402 and SMMC-7721 xenografts to oxaliplatin [191]. miR-125b was downregulated in oxaliplatin-resistant HCC tissues and its overexpression facilitated HepG2 and SK-Hep-1 xenografts to overcome oxaliplatin resistance by targeting EVA1A, a lysosomal and endoplasmic reticulum-associated protein regulating autophagy and apoptosis [192]. Oxaliplatin and 5-FU induced HULC expression and HULC-mediated protective autophagy provided resistance to chemotherapy [35].

## 8. Conclusions

Hepatocellular carcinoma (HCC) develops following a long-standing chronic inflammatory process, in response to HBV or HCV infection or other insults, such as NASH or aflatoxin, which leads to extensive fibrosis and eventual cirrhosis. This destructive process profoundly compromises liver function, such as metabolism and drug detoxification, and creates a unique problem for HCC patients not faced by most patients from other cancers with a functioning liver. HCC patients are profoundly resistant to conventional chemo- and radiotherapy and they are highly sensitive to drug-induced toxicity because of loss of liver function. Consequently, drug compliance by patients is reduced contributing further to the lack of therapeutic efficacy of the drugs. In this scenario drug-based therapies have less chances to be successful in HCC patient management. Gene-based therapies provide a better alternative especially because of high payload delivery to the target organ liver following systemic administration. ncRNAs have the potential to have strong impact in HCC treatment because AgomiRs or antagomiRs can be efficiently delivered to the liver by targeted nanoparticles. They are relatively non-toxic, and because of their size, they have less chance to induce an immune response. A phase 1 study with MRX34, a liposomal miR-34a mimic, showed manageable toxicity profile in most patients and some clinical activity in HCC patients [193]. Although the study needed to be terminated because of serious adverse effects in some patients, it established the proof-of-concept for miRNA-based therapy. One caveat of this study is that, although miR-34 functions as a tumor suppressor for most cancers, recent studies indicate that it might have oncogenic function in specific contexts of HCC, and inhibition of miR-34a using a locked nucleic acid (LNA) effectively abrogated the HCC progression rate in mice with β-catenin activation [194,195]. It would be interesting to determine the efficacy of a liver-targeted delivery of miR-122, which has been confirmed as an HCC-specific tumor suppressor using knockout mouse models. Many ncRNAs, especially miRNAs, are released into the circulation by tumor cells via exosomes and can serve as potential diagnostic and prognostic markers for HCC and indeed, following pre-clinical studies, several clinical trials are currently ongoing with that aim in view, such as NCT02448056 (miRNA as a diagnostic and prognostic biomarker of hepatocellular carcinoma). It is expected that, in the coming years, ncRNAs will have more prominent roles in clinical management of HCC patients, including diagnosis, treatment, and treatment response. For this purpose, more in-depth studies are required with proper mouse models to determine the functions of the ncRNAs, both in physiology and in disease process, and to unravel their molecular mechanisms of action to predict potential consequences of perturbing them during the disease process.

## Figures and Tables

**Table 1:** Examples of dysregulated long non-coding RNAs (lncRNAs) in hepatocellular carcinoma (HCC).

Clinical Samples Used	ncRNA	Genomic Location	Expression Level	Function	References
63 HCC tissues and corresponding adjacent healthy tissues [16]	HOTAIR	12q13.13	Up-regulated	Enhances EMT and tumorigenesis by interacting with EZH2 to downregulate miR-218 and by functioning as sponge for a number of microRNAs (miRNAs), such as miR-23b-3p, miR-145, and miR-122	[16,17,18,19,20,21,22]
56 pairs of HCC and corresponding non-HCC tissues [27]	MALAT1	11q13.1	Up-regulated	Interacts with SRSF1 to activate mTOR signaling, increases TCF7L2 translation to promote glycolysis, sponges miR-195, miR-143-3p, and miR-146-5p to promote HCC and induce therapy resistance	[24,25,26,27,28,66]
46 HCC tissues, 4 FNH, 7 cirrhosis and 2 normal liver [29]	HULC	6p24.3	Up-regulated	Silences miR-9 to promote lipogenesis, silences EEF1E1, upregulates CLOCK and SIRT1, sponges a number of miRNAs, such as miR-2001-3p and miR-186	[29,30,31,32,33,34,35]
64 HCC tissues [36]	H19	11p15.5	Up-regulated	Sponges miR-193b to promote EMT and stem cell transformation	[36,37]
52 HCC needle biopsies and matched non-tumor tissue [39]	HOTTIP	7p15.2	Up-regulated	Stimulates transcription of Hox genes, upregulates GLS1	[39,40]
95 pairs of HCC and adjacent non-cancerrous liver [47]	NEAT1	11q13.1	Up-regulated	Facilitates HCC by sponging several miRNAs, such as miR-485, miR-204 and miR-139-5p	[45,46,48]
50 paired HCC and non-HCC samples [41]	HEIH	5q35.3	Up-regulated	Interacts with EZH2 to silence p16	[41]
56 pairs of HCC tumor tissues and adjacent normal tissues [49]	TINCR	19p13.3	Up-regulated	Sponges miR-218-5p to upregulate Deadbox helicase 5 (DDX5) and activate AKT	[49]
48 HCC and matched adjacent non-malignant Tissues [50]	SNHG5	6q14.3	Up-regulated	Promotes HCC progression by sponging miR-26a-5p and modulating Glycogen synthase kinase 3 β (GSK3β) and Wnt/β-catenin pathways	[50]
84 pairs of HCC and corresponding peritumor tissues [51]	HCAL	4q26	Up-regulated	Facilitates HCC by sponging miR-15a, miR-196a and miR-196b and upregulating Lysosomal protein transmembrane 4 β (LAPTM4B)	[51]
23 pairs of HCC and adjacent non-HCC samples [52]	MEG3	14q32.2	Down-regulated	Acts as tumor suppressor by interacting with p53 and sponging miR-664	[52,54,55,56]
50 HCC patient samples [57]	GAS5	1q25.1	Down-regulated	Sponges miRNAs and inhibits vimentin expression	[57,58,59]
30 paired HCC and matched normal tissues [61]	FENDRR	16q24.1	Down-regulated	Inhibits GPC3 by promoter methylation, sponges miR-423-5p to upregulate GADD45B	[61,62]
195 pairs of HCC and corresponding peri-tumor tissues [63]	DILC	13q34	Down-regulated	Interacts with IL-6 promoter to block IL-6/STAT3 signaling	[63]
170 human HCC Samples and adjacent tissues [64]	uc.134	3	Down-regulated	Interacts with Cullin 4A (CUL4A) to inhibit ubiquitination of Large tumor suppressor kinase 1 (LATS1) and silence Yes1 associated transcriptional repressor (YAP)	[64]
38 HCC samples and adjacent non-tumor tissue; 129 HCC samples [65]	lnc-FTX	Xq13.2	Down-regulated	Binds to replication factor Minichromosome maintenance complex component 2 (MCM2) to prevent DNA replication and sponges miR-374a activating Wnt/β-catenin signaling	[65]

**Table 2:** Examples of deregulated expression of small ncRNAs in HCC.

Clinical Samples Used	ncRNA	Genomic Location	Expression Level	Function	References
21 normal livers, 104 HCC, 90 paired cirrhotic tissues and 35 HCC-derived cell lines [80]	miR-21	17q23.2	Up-regulated	Targets many tumor suppressor genes, most importantly PTEN activating PI3K/AKT pathway	[80,81,82,83,84]
21 normal livers, 104 HCC, 90 paired cirrhotic tissues and 35 HCC-derived cell lines [80]	miR-221	Xp11.3	Up-regulated	Targets many tumor suppressor genes, such as p27, p57 that regulate cell cycle	[80,85,86,87,88,89]
20 HCC tissues and pair-matched normal liver tissues [90]	miR-155	21q21.3	Up-regulated	Targets APC to activate Wnt/β-catenin pathway and SOCS1 to activate STAT3 signaling, as well as C/EBPβ	[90,91,92,93]
17 HCC and 21 cirrhotic liver tissues [76]	miR-122	18q21.31	Down-regulated	Targets many genes regulating lipid metabolism, inflammation and fibrosis contributing to steatohepatitis and HCC	[76,94,95,96,97]
17 pairs of HCC and adjacent normal liver [102]	miR-29	7q32. 3	Down-regulated	Promotes apoptosis By targeting Mcl-1 and Bcl-2, changes DNA methylation by targeting DNMT3A	[102,103]
38 HCC and paired normal liver samples [104]	miR-101	1p31.3	Down-regulated	Targets many oncogenes, such as Mcl-1, JunB, Rock2	[104,105,106,107]
20 paired HCC and non-tumor tissues [109]	let-7 family	Multiple chromosomes	Down-regulated	Targets RAS, STAT3, many genes regulating fibrosis	[109,110,111,112]
14 paired HCC and non-tumor tissues [114]	miR-15 family	17p13.1	Down-regulated	Targets IKKα and TAB3 to inhibit NF-κB, VEGF to inhibit angiogenesis	[114,115,116]
30 HCC and 28 liver samples [119]	SNORD126	14q11.2	Up-regulated	Activates PI3K–AKT pathway through FGFR2	[119]
13 pair-matched HCC and normal tissues [124]	SNORA24	4q26	Down-regulated	Perturbation of ribosomal function	[124]
112 pairs of human HBV-associated HCC and adjacent non-tumor tissues [125]	SNORD113-1	14q32	Down-regulated	Suppresses HCC tumorigenesis in MAPK/ERK and TGF-β pathway-dependent mechanisms	[125]
73 pairs of HCC and adjacent non-tumor tissue [129]	piR-Hep1	1q24.2	Up-regulated	Promote cell proliferation and invasion via activating PI3K/AKT signaling pathway	[129]

**Table 3:** Examples of deregulation of circular RNAs (circRNAs) in HCC.

Clinical Samples Used	ncRNA	Expression Level	Function	References
289 HCC samples and paired adjacent liver tissues	circMTO1	Down-regulated	Acts as a sponge for oncogenic miR-9 to promote p21 expression	[133]
208 pairs of HCC and adjacent normal liver	cSMARCA5	Down-regulated	Sponges miR-17-3p and miR-181b-5p which target TIMP3	[134]
75 HCC tissues with metastasis or no metastasis	circ-10720	Up-regulated	Sponges several miRNAs targeting vimentin and induces EMT and metastasis	[135]
100 paired HCC tissues and adjacent normal tissues	circMAT2B	Up-regulated	Sponges miR-338-3p thus upregulating PKM2 and glycolysis	[136]
15 metastatic and 15 non-metastatic HCC	circASAP1	Up-regulated	Sponges miR-326 and miR-532-5p to induce MAPK1 and CSF-1	[137]
100 paired HCC tissues and adjacent normal tissues	circRHOT1	Up-regulated	Recruits TIP60 to NR2F6 promoter to increase transcription	[138]
